# Assessment of drug use pattern using WHO prescribing indicators at Hawassa University teaching and referral hospital, south Ethiopia: a cross-sectional study

**DOI:** 10.1186/1472-6963-13-170

**Published:** 2013-05-07

**Authors:** Anteneh Assefa Desalegn

**Affiliations:** 1 Anteneh Assefa Desalegn, Pharmacology Unit, School of Medicine, Hawassa University, Hawassa, Ethiopia

**Keywords:** Rational or irrational drug use, WHO core drug use indicators, Prescribing pattern

## Abstract

**Background:**

To promote rational drug use in developing countries, it is important to assess drug use pattern using the World Health Organization (WHO) drug use indicators. The aim of this study was to assess the drug prescription patterns at the Medical Outpatient Pharmacy of Hawassa University Teaching and Referral Hospital, using some of the WHO core drug use indicators.

**Methods:**

A descriptive, quantitative, and cross-sectional survey was conducted to determine the current prescribing practices at Hawassa University Teaching and Referral Hospital. The sample was selected using systematic random sampling. 1290 patient encounters were reviewed retrospectively for a 2-year period from September 2007 to September 2009. Data were collected from prescriptions and Prescription registration books retained in the pharmacy.

**Result:**

The average number of drugs prescribed per encounter or mean was 1.9 (SD 0.91) with a range between 1 and 4. The percentage of encounters in which an antibiotic or injection was prescribed was 58.1% (n = 749) and 38.1% (n = 491), respectively. The Percentage of drugs prescribed by generic name and from an essential drug list was 98.7% (n=2419) and 96.6% (n=2367), respectively. The most commonly prescribed forms of antibiotics were amoxicillin (16.4%), ampicillin (15%), gentamicin (14.9%) and chloramphenicol (11.6%). On the other hand, the most commonly prescribed injections were ampicillin (21.4%), cloxacillin (13.4%), crystalline penicillin (12.4%), ceftriaxon (9.8%) gentamicin (9.8%), diclofenac (9.4%), chloramphenicol 41 (8.4%) and furosemide 25 (5.1%).

**Conclusion:**

On the basis of the finding of this study, the prescribing practices for antibiotic and injection shows deviation from the standard recommended by WHO. These two commonly overused and costly forms of drug therapy need to be regulated closely. Drug use evaluation should be done for some of the antibiotics to check whether they were appropriately prescribed or not. On the other hand, polypharmacy, generic prescribing and prescribing from EDL were not found to be a problem in this study. Teaching hospitals have a special responsibility to society to promote rational prescribing by their staff and, through them, the future generations of doctors.

## Background

The rational use of drugs requires that “patients receive medications appropriate to their clinical needs, in doses that meet their own individual requirements for an adequate period of time, at the lowest cost to them and their community” [1]. The irrational use of drugs is a problem, and to manage it the World Health Organization (WHO) convened an international conference in Nairobi, Kenya, in 1985 to develop useful guidelines [1,2].

Essential drugs comprise one of the tools needed to fight ill health. By increasing access to essential drugs and their rational use, we could improve health status and secure development gains [3]. “Essential drugs are those that satisfy the health care needs of the majority of the population; they should therefore be available at all times, in adequate amounts and in the appropriate dosage forms” [4]. This concept was introduced to accelerate the positive impacts of drugs on health status, particularly for developing countries [3,5].

Worldwide, more than half of all medicines are prescribed, dispensed, or sold improperly, and 50% of patients fail to take them correctly. Moreover, about one third of the world’s population lacks access to essential medicines [6]. A survey conducted in 8 hospitals in southern Ethiopia that investigated their prescription patterns concluded that irrational prescribing, as evidenced by high average number of drugs prescribed per encounter, high percentage of injections, and high percentage of antibiotic use, was prevalent in the studied region [7]. However, another study found good signs of rational drug prescribing at Jimma Hospital, south west Ethiopia [8].

Irrational prescribing is a global problem. Bad prescribing habits lead to ineffective and unsafe treatment, exacerbation or prolongation of illness, distress and harm to the patient, and higher costs. Irrational prescribing patterns are perpetuated through patient pressure, bad example of colleagues, and high-powered salesmanship by drug company representatives. In teaching hospitals, new graduates will copy them, completing the vicious circle. Changing existing prescribing habits becomes very difficult [2].

Assessment of drug use patterns with the WHO drug use indicators is becoming increasingly necessary to promote rational drug use in developing countries [9,10]. Before activities are started to promote rational drug use, an effort should be made to describe and quantify the situation. Several well-established survey methods are available for this purpose. One assessment method is a prescribing and patient care survey using the WHO health facility drug use indicators. These quantitative indicators are now widely accepted as a global standard for problem identification and have been used in over 30 developing countries [11].

This study assessed the drug prescribing patterns in the outpatient pharmacy of Hawassa University Teaching and Referral Hospital by using some of the WHO core drug use indicators.

## Method

### Study setting

Hawassa is located in the southern part of Ethiopia. The study was conducted at Hawassa University Teaching and Referral Hospital, in Hawassa, which is about 270 km south of Addis Ababa. It is the main referral hospital in southern region.

### Study design

A retrospective, quantitative, and cross-sectional survey designed to describe the current prescribing practices at Hawassa University Teaching and Referral Hospital.

### Data collection and analysis

Three well-trained pharmacy personnel collected data on prescribing indicators retrospectively by using prescriptions and prescription registration books. The specific types of data necessary to measure the prescribing indicators were recorded for each patient encounter and entered directly into an ordinary prescribing indicator form.

According to the WHO document “How to investigate drug use in health facilities,” at least 600 encounters should be included in a cross-sectional survey to describe the current prescribing practices, with a greater number, if possible [10]. For this particular study, more than 1,200 prescriptions were collected retrospectively from more than 50,000 prescriptions written for a 2-year period from September 2007 to September 2009. This indicator study is also restricted to a sample of general illness encounters, representing a mix of health problems and patient ages. The sample was selected using a systematic random sampling method, and the sampling unit was patient encounters taking place at the outpatient health facility for the treatment of acute and chronic illness.

All data in the ordinary prescribing indicator recording form were first analyzed manually and then using Microsoft Excel 2007. In the statistical analysis, frequencies, averages/means, standard deviations and percentages were obtained.

### Prescribing indicators

The WHO prescribing indicators were used in this study. The indicators were pretested, and slight modification was made so that they could be used easily to provide accurate data. The final versions of the pretested indicators are described below.

The prescribing indicators that were measured included:

1. The average number of drugs prescribed per encounter was calculated to measure the degree of polypharmacy. It was calculated by dividing the total number of different drug products prescribed by the number of encounters surveyed. Combinations of drugs prescribed for one health problem were counted as one.

2. Percentage of drugs prescribed by generic name is calculated to measure the tendency of prescribing by generic name. It was calculated by dividing the number of drugs prescribed by generic name by total number of drugs prescribed, multiplied by 100.

3. Percentage of encounters in which an antibiotic was prescribed was calculated to measure the overall use of commonly overused and costly forms of drug therapy. It was calculated by dividing the number of patient encounters in which an antibiotic was prescribed by the total number of encounters surveyed, multiplied by 100.

4. Percentage of encounters with an injection prescribed was calculated to measure the overall level use of commonly overused and costly forms of drug therapy. It was calculated by dividing the number of patient encounters in which an injection was prescribed by the total number of encounters surveyed, multiplied by 100.

5. Percentage of drugs prescribed from an essential drug list (EDL) was calculated to measure the degree to which practices conform to a national drug policy as indicated in the national drug list of Ethiopia. Percentage is calculated by dividing number of products prescribed which are in essential drug list by the total number of drugs prescribed, multiplied by 100.

### Operational definitions

Generic drugs: The essential drug list of Ethiopia is used as a basis to determine drugs as generic or brand name.

Antibiotics: Drugs such as penicillins, antibacterials, anti-infective dermatological drugs, and anti-infective opthalmological agents, antidiarrheal drugs with streptomycin, neomycin, and metronidazole are considered antibiotics when used in the context of antibiotics.

Combination of drugs: Two or more drugs that are prescribed for a given health condition. For example, triple therapy for helicobacter pylori induced peptic ulcer is counted as one.

### Ethical consideration

Ethical approval was obtained from the Hawassa University Institutional Review Board.

## Results

A sample of 1290 patient encounters was assessed retrospectively in the medical outpatient pharmacy of Hawassa University Hospital from September 2007 to September 2009.

A total of 2451 drug products were prescribed. Thus, the average number of drugs per prescription or mean was 1.9 (SD 0.91) with a range between 1 and 4. The total number of drugs prescribed by generic name was 2419 (98.7%). An antibiotic was prescribed in 749 patient encounters (58%), and an injection was prescribed in 491 encounters (38.1%) (Table 1). Almost all drugs prescribed (n = 2367, 96.6%) were on the essential drug list of Ethiopia.

**Table 1:** Summary of results obtained at Hawassa University Hospital from September 2007 to September 2009 (n = 1290 encounters)

**Prescribing indicators assessed**	**Total drugs/ encounters**	**Average/ percent**	**Standard derived or ideal**
Average number of drugs per encounter	2451	1.9	(1.6-1.8)
Percentage of encounter with antibiotics	749	58.1%	(20.0-26.8%)
Percentage of encounters with injection	491	38.1%	(13.4%-24.1%)
Percentage of drugs prescribed by generic	2419	98.7%	100%
Percentage of drugs from essential drug list	2367	96.6%	100%

Of a total of 2451 drugs prescribed, 841 (34.3%) were antibiotics. The most commonly prescribed antibiotics were amoxicillin 138 (16.4%), ampicillin 126 (15%), gentamicin 125 (14.9%), chloramphenicol 98 (11.6%), cloxacillin 71 (8.4%), crystalline penicillin 61 (7.3%), ciprofloxacin 57 (6.7%), ceftriaxone 48 (5.9%), and doxycycline 47 (5.6%) (Table 2).

**Table 2:** Most commonly prescribed antibiotics at the medical outpatient pharmacy of Hawassa University Referral Hospital from September 2007 to September 2009

**Commonly prescribed antibiotics**	**Frequency**	**Percentage (%)**
Amoxicillin	138	16.4
Ampicillin	126	15
Gentamicin	125	14.9
Chloramphenicol	98	11.6
Cloxacillin	71	8.4
Crystalline penicillin	61	7.3
Ciprofloxacin	57	6.7
Ceftriaxone	48	5.9
Doxycycline	47	5.6
Cotrimoxazole	38	4.5
Erythromycin	14	1.6
Cephalexin	10	1.2
Metronidazole	8	0.9
Total	841	100

The percentage of encounters in which an injection was prescribed at Hawassa University Hospital was 38.1%. The most commonly prescribed injections were ampicillin 105 (21.4%), cloxacillin 66 (13.4%), crystalline penicillin 61 (12.4%), ceftriaxon 48 (9.8%) gentamicin 48 (9.8%), diclofenac 46 (9.4%), chloramphenicol 41 (8.4%) and furosemide 25 (5.1%) (Table 3).

**Table 3:** Most commonly prescribed injections at the medical outpatient pharmacy of Hawassa University Referral Hospital from September 2007 to September 2009

**Commonly prescribed injection**	**Frequency**	**Percentage (%)**
Ampicillin	105	21.4
Cloxacillin	66	13.4
Crystalline Penicillin	61	12.4
Ceftriaxone	48	9.8
Gentamicin	48	9.8
Diclofenac	46	9.4
Chloraphinicol	41	8.4
Furosemide	25	5.1
Dexamethasone	23	4.7
Chlorpromzine	8	1.6
Diazepam	7	1.4
Metrindazole	4	0.8
Total	482	98.2

## Discussion

The average number of drugs per prescription, 1.9, at Hawassa University Hospital is acceptable compared with the standard (1.6-1.8) derived as ideal [12]. In a similar study performed in south west Ethiopia at Jimma Hospital, the average number of drugs per encounter was 1.59, which was also in the acceptable range [8]. However, in a study on prescribing patterns in three hospitals in north Ethiopia, the average number of drugs per patient was 0.98 at Gondar Hospital, 1.8 in Bahirdar Hospital, and 2.2 in Debre Tabor Hospital [13]. A national baseline study on drug use indicators in Ethiopia in September 2002 also found the average number of drugs prescribed per encounter to be 1.9, which is similar to our finding [14]. In the study of drug use patterns in 12 developing countries, the average number of drugs per encounter was high in Nigeria (3.8), low in Sudan (1.4), and in Zimbabwe (1.3)[15-17]. A high average number of drugs might be due to financial incentives to prescribers to prescribe more, lack of therapeutic training of prescribers, or shortage of therapeutically correct drugs. The low values might mean there is constraint in the availability of drugs, or prescribers have appropriate training in therapeutics.

The percentage of drugs prescribed by generic name at Hawassa University Hospital, 98.7%, is almost similar with the standard derived to serve as ideal (100%) [12]. In a similar study carried out at Jimma Hospital, south west Ethiopia, the percentage of drugs prescribed by generic name was 75.2%, which is low compared to the standard and to our finding [8]. A national baseline study on drug use indicators in Ethiopia in September 2002 also showed the percentage of drugs prescribed by generic name to be 87%, which is lower than our finding of 98.7% [14]. In the study of 12 developing countries, the percentage of generic drugs prescribed was low in Nigeria (58%) and Sudan (63%) but was encouraging in Tanzania (82%) and Zimbabwe (94%) [15-18].

The percentage of encounters in which antibiotics were prescribed at Hawassa University Hospital was 58%, which is very high compared to the standard (20.0%-26.8%) derived to be ideal [12]. This finding suggests that antibiotic prescribing needs to be regulated. The high percentage of antibiotics prescribed in our study setting may be due to cultural beliefs about antibiotics, patient expectation to receive antibiotics, or prescribers’ belief that the therapeutic efficacy of antibiotics is low. It might also have been prescribed appropriately as our setting is a referral hospital where the prescribing pattern is complex. Drug use evaluation should be done to evaluate whether the antibiotics were prescribed appropriately or not. A national baseline study on drug use indicators in Ethiopia in September 2002 also showed that the percentage of encounters in which an antibiotic was prescribed to be 58.1%, which was similar to our finding [14]. In the drug use pattern study in 12 developing countries, the percentage of encounters in which an antibiotic was prescribed was high in Sudan (63%), Uganda (56%), and Nigeria (48%) and relatively better in Zimbabwe (29%) [15-19].

The percentage of encounters in which an injection was prescribed at Hawassa University Hospital was 38.1%, which is higher than the standard (13.4%-24.1%) derived to serve as ideal [12]. Possible reasons for the high use of injections could be (i) beliefs and attitudes of patients and health professionals about the efficacy of injection versus oral medication or (ii) our study setting is a referral hospital where patients with serious conditions are treated, and injectable forms produce faster onset of action. A national baseline study on drug use indicators in Ethiopia in September 2002 found the percentage of encounters with an injection to be 23%, which is lower than our finding (38.1%) and in the acceptable range [14]. In a prescription pattern study in 12 developing countries, the percentage of encounters in which an injection was prescribed was high in Uganda (48%) and Sudan (36%) but very low in Zimbabwe (11%), and in the acceptable range in Indonesia (17%), Ecuador (17%), and Mali (19%) [15-20]. Injections are very expensive compared to other dosage forms and require trained personnel for administration. Moreover, unhygienic use of injections can increase the risk of transmission of potentially serious pathogens, such as hepatitis, HIV/AIDS, and blood-borne diseases.

The percentage of drugs prescribed from the essential drug list for Hawassa University Hospital in the study period, September 2007 to December 2009, was 96.6%, which is almost identical with the standard (100%) derived to serve as ideal [12]. A study of the patterns of prescription at Jimma Hospital, south west Ethiopia, showed similar results, where almost all drugs prescribed for the health problems were on the essential drug list of the country, but few drugs prescribed out of the list were those that were on the national drug list of Ethiopia [8]. A national baseline study on drug use indicators in Ethiopia in September 2002 showed that the percentage of drugs prescribed from the essential drug list to be 99%, which is very encouraging [14]. In a study of prescription patterns from 12 developing countries, the percentage of drugs prescribed from the essential drug list was 88% in Tanzania and 96% in Nepal [18,20]. The EDL of Ethiopia can be accessed from Food, Medicine and Health Care administration and control Authority of Ethiopia (FMHACA) [21].

The most commonly prescribed drugs in Ethiopia are analgesics and antibiotics. Overuse of antibiotics (38%) is an indication of a problem because it could facilitate emergence of resistance. Moreover, the cost incurred is high due to extravagant prescribing where drugs are prescribed for viral infection or for infections in which symptomatic treatment is enough. In addition, empirical treatment is also a problem, where two or more drugs are prescribed but one specific antibiotic is enough after proper diagnosis.

## Limitations

The study used the WHO prescribing indicators, which are supposed to record exactly what is prescribed to patients, but not why. In order to explain why, other techniques are needed. The prescribing indicators measure aspects of outpatient treatment. They are designed for use in health centers, dispensaries or hospital outpatient departments. The prescribing indicators are less useful in specialty outpatient clinics in referral hospitals where the drug use pattern is more complex.

## Conclusion

On the basis of the finding of this study, the prescribing practices for antibiotic and injection shows deviation from the standard recommended by WHO. These two commonly overused and costly forms of drug therapy need to be regulated closely. Drug use evaluation should be done for some of the antibiotics to check whether they were appropriately prescribed or not. On the other hand, polypharmacy, generic prescribing and prescribing from EDL were not found to be a problem in this study. Baseline data gathered by this study can be used by researchers and policymakers to improve prescribing practice at Hawassa University Hospital.

Several activities have proved useful and effective in promoting rational drug use and should be recommended for general use. These are standard treatment guidelines; essential drug lists; establishing drug and therapeutic committee; problem-based basic training in pharmacotherapy; targeted continuing education; availability, accessibility, and affordability of drugs of a good standard; drug information centers; drug use evaluation and drug bulletins. Care is, of course, necessary to implement and ensure success.

## Abbreviations

AIDS: Acquired immunno deficiency syndrome; EDL: Essential drug list; HIV: Human immune deficiency virus; NPT: Netherlands Programme for Institutional Strengthening of Post-secondary Education and Training Capacity; RDU: Rational drug use; SD: Standard deviation; WHO: World health organization

## Competing interest

As the author of this manuscript, I declare that I have no competing interests.

## Pre-publication history

The pre-publication history for this paper can be accessed here:

http://www.biomedcentral.com/1472-6963/13/170/prepub
